# Germline *PALB2* Mutation in High-Risk Chinese Breast and/or Ovarian Cancer Patients

**DOI:** 10.3390/cancers13164195

**Published:** 2021-08-20

**Authors:** Ava Kwong, Vivian Y. Shin, Cecilia Y. S. Ho, Aleena Khalid, Chun Hang Au, Karen K. L. Chan, Hextan Y. S. Ngan, Tsun-Leung Chan, Edmond S. K. Ma

**Affiliations:** 1 Department of Surgery, The University of Hong Kong, Hong Kong, China; vyshin@hku.hk (V.Y.S.); u3549370@connect.hku.hk (A.K.); 2 University of Hong Kong-Shenzhen Hospital, Hong Kong, China; 3 Department of Surgery, Hong Kong Sanatorium & Hospital, Hong Kong, China; 4 Hong Kong Hereditary Breast Cancer Family Registry, Hong Kong, China; Chris.TL.Chan@hksh.com (T.-L.C.); eskma@hksh.com (E.S.K.M.); 5 Department of Pathology, Division of Molecular Pathology, Hong Kong Sanatorium & Hospital, Hong Kong, China; cecilia.ys.ho@hksh.com (C.Y.S.H.); Tommy.CH.Au@hksh.com (C.H.A.); 6 Department of Obstetrics and Gynecology, The University of Hong Kong, Hong Kong, China; kklchan@hku.hk (K.K.L.C.); hysngan@hku.hk (H.Y.S.N.)

**Keywords:** hereditary breast cancer, *PALB2* mutation, Chinese, breast cancer risk

## Abstract

**Simple Summary:**

Breast cancer is the most commonly diagnosed cancer in women globally. *BRCA1* and *BRCA2* mutations are most prevalent in hereditary breast cancer and are associated with increased risk of breast and ovarian cancer. The *PALB2* (partner and localizer of BRCA2) mutation was found to be associated with an increased risk of breast cancer and one of the most common mutations, after *BRCA1* and *BRCA2*, in non-*BRCA1/2* breast cancer patients. The prevalence of the *PALB2* mutation in breast cancer varies across different ethnic groups; hence, it is of intense interest to evaluate the cancer risk and clinical association of *PALB2* mutation in Chinese breast and/or ovarian cancer patients.

**Abstract:**

The prevalence of the *PALB2* mutation in breast cancer varies across different ethnic groups; hence, it is of intense interest to evaluate the cancer risk and clinical association of the *PALB2* mutation in Chinese breast and/or ovarian cancer patients. We performed sequencing with a 6-gene test panel (*BRCA1, BRCA2, TP53*, *PTEN*, *PALB2*, and *CDH1*) to identify the prevalence of the *PALB2* germline mutation among 2631 patients with breast and/or ovarian cancer. In this cohort, 39 mutations were identified with 24 types of mutation variants, where the majority of the mutations were frame-shift mutations and resulted in early termination. We also identified seven novel *PALB2* mutations. Most of the *PALB2* mutation carriers had breast cancer (36, 92.3%) and were more likely to have family history of breast cancer (19, 48.7%). The majority of the breast tumors were invasive ductal carcinoma (NOS type) (34, 81.0%) and hormonal positive (ER: 32, 84.2%; PR: 23, 60.5%). Pathogenic mutations of *PALB2* were found in 39 probands with a mutation frequency of 1.6% and 1% in breast cancer and ovarian cancer patients, respectively. *PALB2* mutation carriers were more likely have hormonal positive tumors and were likely to have familial aggregation of breast cancer.

## 1. Introduction

Breast cancer is a growing public health concern, as it is the most commonly diagnosed cancer in women globally. Inherited gene mutation is one of the risk factors for developing breast cancer or ovarian cancer in women. Over the past decades, a number of mutations have been identified in hereditary breast cancer, among which *BRCA1* and *BRCA2* mutations are the most prevalent and associated with increased risk of breast and ovarian cancer [1]. Genetic testing is becoming more important, since the test results greatly impact the clinical management of high-risk patients and their family members. With the emergence of next-generation sequencing (NGS), utilization of multigene panel with high-penetrance genes (e.g., *BRCA*) and moderate- or low-penetrance genes are included for cancer diagnosis. The *PALB2* (partner and localizer of BRCA2) mutation was found to be associated with an increased risk of breast cancer and one of the most common mutations, after *BRCA1* and *BRCA2*, in non-*BRCA1/2* breast cancer patients [2,3,4,5]. The clinical evidence of *PALB2* variants is increasing, and the clinical guidelines and management for *PALB2* mutation carriers are mainly based on consensus but still lack solid supportive evidence. Hence, additional studies that allow the mutation spectrum and the clinical outcomes of *PALB2* mutation carriers to be understood has implication for public health.

The *PALB2* gene encodes for a tumor suppressor protein that binds to and co-localizes with BRCA2 in the nucleus and assists in the formation of the BRCA1–PALB2–BRCA2 complex [6]. It is Fanconi anemia (FA) gene located on 16q12.22, which plays a key role in double strand break repair by homologous recombination alongside *BRCA1/2* [7]; hence, mutations in *PALB2* lead to defects in DNA repair pathways. Biallelic mutations in *PALB2* gene lead to FA subtype-D1 (FA-D1) and childhood solid tumors, whereas the monoallelic mutation has been linked to hereditary breast and ovarian cancer syndrome [8,9]. The lifetime risk of developing breast cancer in women with the *PALB2* mutation is 52.8% by age of 80 [3].

The prevalence and frequency of the *PALB2* mutation in breast cancer varies across different ethnic groups [10]. According to a study performed in the Finnish population, *PALB2* c.159delT was identified as the founder mutation in the respective population [11]. Similarly, a *PALB2* screening study performed in 1403 female Australian breast cancer patients followed by screening of 779 families revealed that *PALB2* c.3113G>A was the most recurrent familial mutation [12]. Moreover, *PALB2* mutations accounted for 1.1% of the breast cancer cases in the Caucasian population [13]. Our recent study reported that the mutation frequency of *PALB2* was 1.4%, which was the most frequently mutated gene after *BRCA1* and *BRCA2* genes in Chinese patients [14]. Similarly, studies showed that *PALB2* mutations account for approximately 1–2% of early onset breast cancer patients in the Chinese population [10,15]. Thus, understanding the cancer risk and phenotypic presentations of the *PALB2* mutation in Chinese breast cancer contribute to clinical management decisions.

To date, no effective therapeutic strategies have been devised for *PALB2* mutation-carrying breast cancer patients, despite a few case studies suggesting the use of platinum-based chemotherapies in the metastatic setting. Recently, there was a report of a *PALB2* mutation-carrying breast cancer patient treated with carboplatin for 7 months, whom after relapse showed complete response and disease-free post 30-months of treatment [16]. Similarly, another patient with invasive ductal carcinoma, post relapse, upon treatment with carboplatin showed complete response. Unfortunately, due to recurrent thrombocytopenia, the treatment was stopped, and the patient passed away. Another study showed promising results with the use of PARP inhibitor (olaparib) in a breast cancer patient with the *PALB2* mutation [17]. These studies suggest that platinum-based therapies and PARP inhibitors could potentially be used to treat *PALB2* mutation carriers. Better understanding of *PALB2* mutation carriers would allow for better evidence to support clinical guidelines for the management of such carriers. 

## 2. Results

### 2.1. Patients’ Characteristics of the Cohort

Our testing cohort included 2631 patients with breast cancer and/or ovarian cancer. The median age at diagnosis of breast cancer was 43 years (range 18–88) and 49 years (range 9–85) for ovarian cancer. Of these, 2105 (80.0%) were breast cancer patients, 398 (15.1%) were ovarian cancer patients, and 128 (4.9%) were diagnosed with both breast and ovarian cancers. Bilateral breast cancers were seen in 552 patients (24.7%). The majority of the breast cancers were ductal carcinoma (NOS Type) (1872, 69.7%). There was a high percentage of breast cancers, which were found to be hormonal positive (1525, 70.8%) followed by triple-negative breast cancers (TNBC) (430, 20.0%). Most of the breast tumors were diagnosed at early stages (0, I, or II) (2203, 85.0%) and grading favor grade 2 or 3 (858, 45.6% and 722, 38.4%, respectively). Most of the ovarian cancers were diagnosed with epithelial cancers (471, 96.3%) and the majority of high grade (294, 64.2%). A positive family history of breast cancer (first- or second-degree relatives) was seen among 952 patients (36.2%). Family history of ovarian cancer and prostate cancer were also seen in 162 (6.2%) and 123 (4.7%), respectively, of their relatives. Detailed clinicopathological characteristics are shown in Table 1.

### 2.2. Characteristics of Mutation Carriers

Heterozygous pathogenic mutations of *PALB2* were found in 39 probands with a mutation frequency of 1.6% among high-risk breast and 1% of ovarian cancer patients, and there was one patient with double heterozygous mutations identified in *BRCA1* and *PALB2*. Among the single mutation carriers, the median age of breast cancer diagnosis was 39 (range 24–69) and 59 (range 36–65) for ovarian cancer mutation carriers; all of the *PALB2* carriers were female (Table 2). The majority of the cases are patients with breast cancer (36, 92.3%) and 5 out of 39 (12.8%) with ovarian cancer. Four of the *PALB2* carriers had multiple cancers including breast cancer, ovarian cancer, colorectal cancer, or cancer in the uterus. The percentage of bilateral breast cancers in the carriers (27.8%) was similar to the non-carriers (23.5%). A positive family history of breast cancer (first- or second-degree relatives) was seen among 19 of the patients (48.7%). None of them had a family history of ovarian cancers in their relatives. 

Most of the breast tumors were diagnosed with invasive ductal carcinoma (NOS type) (34, 81.0%) of grade 2 (19, 59.4%). Many breast cancers were found to be hormonal positive (ER: 32, 84.2%; PR: 23, 60.5%) with negative expressions of HER2 (25, 86.2%). There were only four (11.4%) triple-negative breast cancers (TNBC). The majority of ovarian cancers were diagnosed with epithelial cancer (3, 100%). Detailed pathological characteristics was shown in Table 3.

There was no significant difference at the age of breast diagnosis and histology between *PALB2* mutation carriers and *BRCA1/2* mutation carriers. Interestingly, all *PALB2* and *BRCA1* mutation carriers were female, and 6.9% of *BRCA2* mutation carriers were men (Table 2). Unlike mutation carriers of *BRCA1/2*, ovarian cancer is not commonly seen in *PABL2* mutation carriers; 26.6% of the *BRCA1* developed personal ovarian cancer, while only 7.7% of *PABL2* carriers developed ovarian cancer (*p*-value < 0.001). Both *PABL2* and *BRCA1/2* mutation carriers had strong family histories of breast cancer. A family history of ovarian cancer was only seen in *BRCA1/2* families but not in *PALB2* families, where 28.3% of *BRCA1* carriers and 12.3% of *BRCA2* carriers had a family history of ovarian cancer (*p*-value = 0.001 and 0.018, respectively). Family history of prostate cancer was seen in 15.4% of *PALB2* carriers but in only 1.7% of *BRCA1* carriers (*p*-value = 0.001); there was no significant different between *PALB2* and *BRCA2* carriers (*p*-value = 0.594). *PALB2* carriers had significant family histories in breast and prostate cancers when comparing with non-carriers (*p*-value = 0.037 and 0.005, respectively). Association of age of ovarian diagnosis and histology between *PALB2* mutation carriers and *BRCA1/2* mutation carriers was not calculated due to limited numbers.

### 2.3. Double Heterozygote in BRCA1 and PALB2

**Figure 1:**
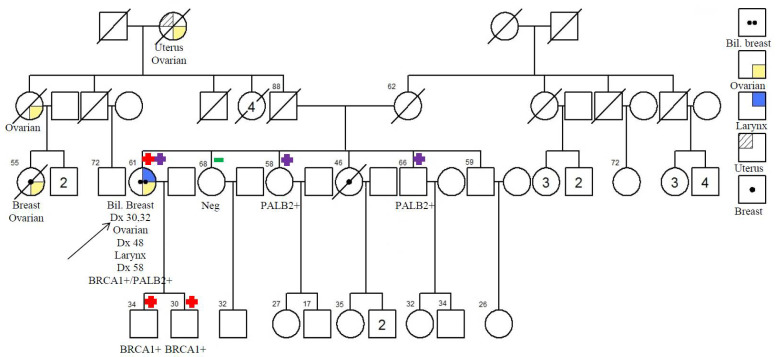
Pedigree of the proband’s family with double heterozygote in *BRCA1* and *PALB2*. 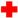 *BRCA1* carrier; 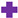 *PALB2* carrier; 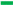 negative; Bil. Breast: bilateral breast.

Double mutations were identified in a 63-year-old female diagnosed with bilateral invasive ductal breast carcinoma of grade 2 and 3, both ER– at the age of 30 and 32 years. She was also diagnosed with FIGO grade 3 epithelial serous ovarian cancer at the age of 48 and cancer at the glottis larynx of T1N0M0 at the age of 58. Detailed family pedigree is shown in Figure 1.

**Figure 2:**
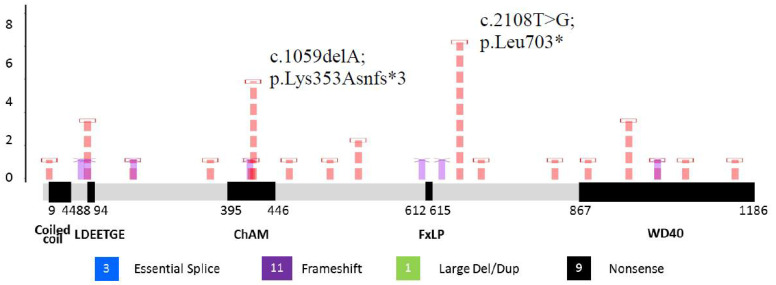
Mutations found in *PALB2*. ChAM: chromatin association motif; LDEETGE: extended EDGE motif; 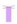 novel mutation; 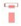 recurrent mutation.

Loss of function variants in *BRCA1* c.3286C>T; p.Gln1096* and a deletion that resulted in frameshift termination c.857delC; p.Pro286Leufs*2 in *PALB2* were identified in this patient (not listed in Figure 2 and Table 3). Mutation c.3286C>T; p.Gln1096* was a base pair alternation in exon 11-9/10 of *BRCA1*, which is not located in any of the reported domains. c.857delC; p.Pro286Leufs*2 in *PALB2* was located in exon 4-3. 

### 2.4. Mutations Specific in PALB2

In this cohort, 39 mutations (1.5%) were identified with 24 types of mutation variants. Eleven (45.8%) of the mutation variants involving a frame-shift resulted in early termination, nine (37.5%) were nonsense mutations, three (12.5%) of the mutations happened at splice sites, and one (4.2%) was a large deletion of exon 4-6 (Table 4). There were seven novel mutations identified (c.181C>T; p.Gln61*, c.212-712_2587-888del9762; p.Pro72Serfs*20, c.448C>T; p.Gln150*, c.1038delA; p.Glu347Asnfs*9, c.1914dupT; p.Glu639*, c.2016dupA; p.Glu673Argfs*42 and c.3201+2T>C; r.3114_3350del237; p.Asn1039_Arg1117del79). There was no specific genomic regional clustering for these mutations in *PALB2*. However, the most frequent mutations (c.2108T>G; p.Leu703* and c.1059delA; p.Lys353Asnfs*3) were seen in seven (17.9%) and five (12.8%) unrelated families, respectively. Details of these mutation variants are shown in Figure 2 and Table 4.

**Figure 3:**
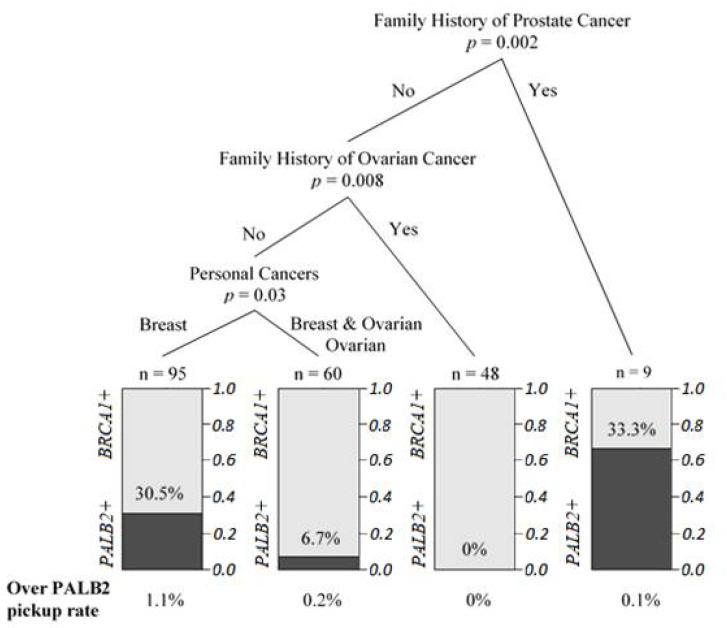
Regression tree for prediction of probability to find *PALB2* pathogenic mutation carriers from *BRCA1* carriers.

**Figure 4:**
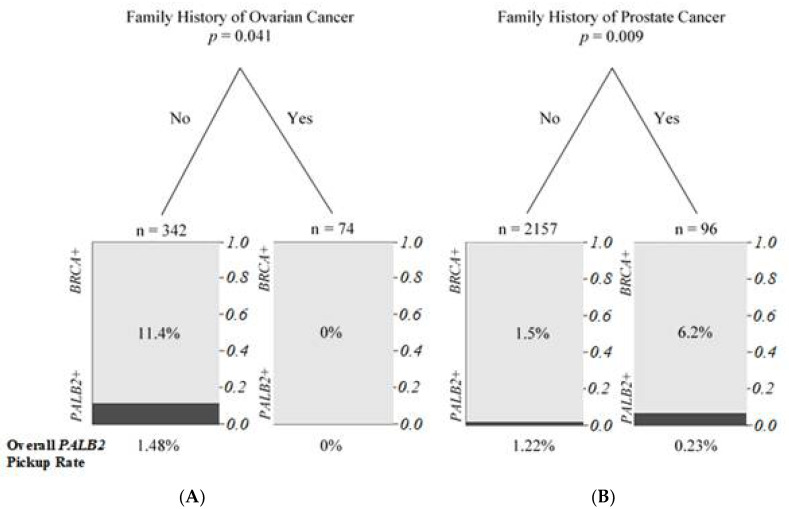
Regression tree for prediction of probability to find *PALB2* pathogenic mutation carriers. (**A**) Regression tree to identify *PABL2* pathogenic carriers from *BRCA1/2* carriers. (**B**) Regression tree to identify *PABL2* pathogenic carriers from mutation negative non-carriers.

Classification and regression tree modelling were performed on the clinicopathological variables to predict the probability of harboring *PALB2* and *BRCA1* mutations. Patients with the *PALB2* mutation were most likely to have personal breast cancer only and no family history of prostate and ovarian cancer when comparing with *BRCA1* carriers (Figure 3). The modelling showed no stratification between *PALB2* and *BRCA2* carriers, which further confirmed that *PALB2* was the partner and localizer of *BRCA2*. As shown in Figure 4, *BRCA1/2* carriers were more likely to have family history of ovarian cancer, but this was not seen in the *PALB2* families. The association with a family history of prostate cancer was seen in *PALB2* carriers compared with mutation negative patients (Figure 4). 

There was no significant difference in disease-free survival probability or overall survival between *PALB2* mutation breast carriers and the mutation-negative breast cancer group, as well as to those with the *BRCA1/2* mutation group in pairwise comparisons (Supplementary Figures S1 and S2). 

## 3. Discussion

*PALB2* has been identified as the third most prevalent breast cancer causing gene with characteristics attributable to germline loss of function monoallelic mutations [6]. According to studies performed in different populations, *PALB2* mutation frequencies in familial breast cancer cases range between 0.6 and 2.7% [1,10,15], while the cumulative average risk reaches approximately 35% by the age of 70, which is similar to the cumulate average risk conferred by germline *BRCA2* mutations [1]. *PALB2* mutation frequency in a Polish cohort was found to be 0.93% [34], 0.8% in the nationwide United States’ population [35], and 0.97% in a Chinese cohort in Mainland China [36].

*PALB2* was believed to contribute moderately to breast cancer risk previously. However, recent studies have identified that the cancer risk is similar to that of *BRCA2* mutations [1]. Family members of patients carrying familial *PALB2* mutations have a 7–9.5-fold increased risk of developing breast cancer with the highest predisposing age group below 40 years [1,3]. A panel study on 34 breast cancer predisposition genes in hereditary breast and ovarian cancer (HBOC) families revealed that the odds ratio of *PALB2* was 10.25 (CI = 6.03–16.40), which was similar to the odds ratio of *BRCA2*, namely 10.26 (CI = 5.75–16.33), therefore deeming it as a high penetrance gene [37,38]. The risk of breast cancer for *PALB2* mutation carriers has been studied worldwide in various populations; the risk of developing breast cancer was six times higher (CI = 2.2–17.2; *p* = 0.01) in Finnish families carrying the c.1592delT founder mutation as compared to the risk of developing non-breast cancer, which was 1.4 (CI = 0.6–3.2; *p* = 0.5) times against normal individuals [39]. In another study performed in a Polish population, two recurrent *PALB2* mutations, c.172_175delGA and c.509_510delGA, were identified at a frequency of 1.5% [40]. Similarly, another *PALB2* pathogenic variant, c.2323C>T was identified in French-Canadian women in Quebec, where the Q775X mutation occurred in about 0.5% of the women diagnosed for breast cancer [41]. An Asian study identified 0.73% and 0.14% in breast cancer and normal control cohorts, respectively, and the most common recurrent mutations were c.7G>T and c.2968G>T in the cancer cohort [19]. A higher risk of developing breast cancer was estimated in *PALB2* mutation carriers, which was 9.1-fold compared to non-carriers in the UK [1]. Another study showed an odds ratio of 2.3 in Australian patients carrying *PALB2* truncating mutations [12]. The absolute risk of breast cancer and male breast cancer were 52.8% and 0.9%, respectively, by age of 80 [3].

Interestingly, we identified a patient with double heterozygous *PALB2* c.857delC and *BRCA1* c.3286C>T mutations who was diagnosed with bilateral breast, ovarian, and larynx cancers. She underwent mastectomy on her right side breast and modified radical mastectomy on her left side breast due to her early age. She also underwent bilateral salpingo-oophorectomy and received chemotherapy, which was well tolerated. She has shown no signs of recurrence after treatment. In the family study, we were only able to obtain the genetic analyses of five of her unaffected cancer-free family members. Her brother and sister both carried only one of the two mutations, namely *PALB2* c.857delC. Both of her sons carried another *BRCA1* c.3286C>T mutation. These findings show that the two mutations are segregated independently (Figure 1). Double heterozygous *PALB2* c.758insT and *BRCA1* c.927delA mutations have also been identified in a German patient with triple-negative breast cancer. She also presented with large myomas of the uterus, a small meningioma and bipolar disorder. However, she has a strong familial aggregation of breast cancer [42]. Another French-Canadian woman with breast cancer was found to harbor *PALB2* c.2323C>T and *BRCA2* c.9004G>A. The maternal side of the double heterozygous carrier contributed to Lynch Syndrome [43].

*PALB2* mutations have been shown to be associated with FA, and patients with FA are sensitive to cross-linking agents [44]. A phase II clinical trial of talazoparib was performed on patients with HER2– breast cancer or other tumors with mutated HR pathway genes, which showed a RECIST response in patients carrying *PALB2* mutations [45]. Similarly, patients with HER2– metastatic breast cancer who received olaparib had an objective response rate of 82% [46]. One patient with two deleterious *PALB2* mutations, one in exon 4, c.1653T>A, and another in exon 6, c.2576C>G, was administered with olaparib and showed a 70% reduction in the tumor burden [47]. Another clinical trial (NCT04756765) based on the use of talazoparib on advanced *PALB2* mutation associated breast cancer is currently underway [48]. Survival data has been studied in different populations, among which Chinese and Polish populations had hazard ratios of 8.38 (CI = 2.19–32.11, *p* = 0.002) and 2.27 (CI = 1.64–3.15; *p* < 0.0001), respectively, thereby with poor overall survival [10,17]. Overall, *PALB2* mutation carriers with breast cancer had a poor 10-year survival rate of 48% with tumors <2cm, while it dropped to 32% for tumors > 2 cm in diameter [10].

Evidence supports that the *PALB2* mutation confers moderate to high breast cancer risk and increased risk for ovarian and pancreatic cancer. The National Comprehensive Cancer Network (NCCN) guidelines (Version 3.2021) has proposed the management of patients with *PALB2* mutations, which includes two strategies [49]. The first is an annual mammogram or consideration of a breast MRI by the age of 30, which may be adjusted to 5–10 years earlier based on the youngest case in the family. Secondly, risk-reducing mastectomy may be performed with respect to residual breast cancer risk and family history. The American College of Medical Genetics and Genomics (ACMG) recommends that *PALB2* gene testing should be included in the test panel for breast, ovarian, and pancreatic cancer. In addition, patients with *PALB2* mutation should be offered breast cancer surveillance similar to that for *BRCA1/2* carriers [50]. Currently, there is no standardized therapy for *PALB2* mutation associated breast cancer due to the limited amount of evidence available; however, some clinical trials have proved the use of PARP inhibitors as beneficial.

The *PALB2* mutation has rarely been found in ovarian cancer patients. The *PALB2* mutation was shown to have an increased risk of ovarian cancer with an odds ratio of 4.6 in a European study. The mutation frequency in epithelial ovarian cancer patients was 0.21% comparing to 0.05% in the control group [51]. In a systematic review of pathogenic *PALB2* mutations, 92.5% of cases described were breast cancer patients, 5.0% of cases were ovarian cancer patients, and 2.4% of cases were pancreatic cancer patients [52].

## 4. Materials and Methods

### 4.1. Ethics Statement

This study was approved by the Institutional Review Board of the University of Hong Kong/Hospital Authority West Cluster and respective authorities of other contributing hospitals in Hong Kong. All participants recruited consented to the study, and the research was conducted according to the Declaration of Helsinki.

### 4.2. Patients

A cohort of 2631 patients with breast and/or ovarian cancer were recruited by the Hong Kong Hereditary and High-risk Breast Cancer Registry between 2012 and 2019 with the following selection criteria: (1) had at least one first- or second-degree relative with *BRCA-*associated cancer, regardless of age; (2) the age at breast cancer diagnosis was under 45 years; (3) bilateral breast cancer; (4) triple-negative breast cancers, (5) cancers with medullary type histology; (6) known to be *BRCA* mutation related family; (7) male breast cancer.

Clinicopathologic characteristics of patient were obtained by medical personnel and medical records (Table 1). Specimens with known *BRCA1/2* mutations (positive control) and anonymous normal volunteer local individuals (negative control) were included for validation of the next generation sequencing (NGS) and to evaluate the performance characteristics of NGS. 

### 4.3. Six-Gene Sequencing Panel

Genomic DNA samples were extracted from peripheral blood samples and subjected to a 6-gene panel (*BRCA1*, *BRCA2*, *TP53*, *PTEN*, *PALB2* and *CDH1*) using our previously-developed protocol [53]. Sequencing libraries from the above-mentioned panels were synthesized and purified as previously described [53,54]. Pooled libraries were loaded onto the MiSeq instrument (Illumina, San Diego, CA) for sequencing. The presence of multi-exonic copy number variations (CNVs) was investigated by computational analysis and multiplex ligation-dependent probe amplification (MLPA). The cDNA from blood was also sequenced in the case of splicing variant analysis at the transcript level. All detected pathogenic variants were verified by the conventional Sanger bi-directional DNA sequencing. The sequencing data was co-analyzed by our in-house developed bioinformatics pipeline.

### 4.4. Variant Interpretation and Annotation

Variants calling bioinformatics was performed as previously described [53,54]. Paired sequencing reads were mapped to the human reference genome sequence GRCh37/hg19. The variants with minor allele frequency of at least 1% reported by the 1000 Genomes Projects [55] were excluded from manual variant curation. Variants were described according to the recommendations of the Human Genome Variation Society (HGVS). Variant descriptions were further verified with Mutalyzer Name Checker (http://mutalyzer.nl, accessed on December 2012 to December 2020).

### 4.5. Statistical Analysis

Clinicopathological variables from pathogenic/likely pathogenic mutation carriers and non-carriers were tabulated in contingency tables. Computation was performed using R (version 3.6.0). Statistical tests suitable for categorical data were then considered. Some variables had expected values of less than 5, and most variables did not have a natural ordering. Fisher’s exact test was adopted. Bonferroni correction was adopted to adjust the significance level for multiple comparison. Conditional Inference Tree (in R package party kit) was also applied to obtain significant factors to predict mutation. Disease-free survival analysis was done on breast cancer patients (mutation carriers of 30 *PALB2*, 93 *BRCA1*, 158 *BRCA2*, and 1695 non-carriers) with surgical information and follow-up data. The survival time was calculated from the date of the first surgery. The next event was defined as the earliest local relapse, distant metastasis, death, or occurrence of 2nd primary cancer. For bilateral cases, if the onset time of 2nd primary was >90 days after 1st primary and before any relapse of 1st primary, the case was treated as censored at the time of 2nd primary diagnosis. The by-gene survival times were then plotted for mutation carriers of 33 *PALB2*, 93 *BRCA1*, 163 *BRCA2*, and 1782 non-carriers. The log-rank test was tested on the significant differences in survival probability. Significance level was set at 5% (*p*-value < 0.05). Disease-free survival analysis and overall survival in ovarian cancer patients were not included due to the limited number of cases.

## 5. Conclusions

We demonstrated that the mutation frequency of *PALB2* was 1.6% among high-risk breast and 1% of ovarian cancer patients. We identified seven novel mutations and two recurrent mutations in *PALB2*. The majority of the *PALB2* mutation tumors were found to be hormonal positive and were likely to have familial aggregation of breast cancer. The mutation screening for *PALB2* should be included in the test panel for breast and ovarian cancer patients. More clinical evidence is needed to demonstrate the effectiveness of PARP inhibitors in patients with *PALB2* mutation, and yet breast cancer surveillance is recommended for these mutation carriers.

## Figures and Tables

**Table 1:** Clinicopathologic characteristics of study cohort.

Clinicopathologic Characteristics	*n* = 2631	%
First diagnosis of breast cancer Mean/Median (Range)	45.1/43 (18–88)	
First diagnosis of ovarian cancer Mean/Median (Range)	48.5/49 (9–85)	
Gender		
Female	2564	97.5%
Male	67	2.5%
Personal Cancer		
Breast Cancer	2105	80.0%
Ovarian Cancers	398	15.1%
Breast and Ovarian Cancers	128	4.9%
Bilateral CA	552	24.7%
Family history of cancers in 1st and 2nd degree		
Breast Cancer	952	36.2%
Ovarian Cancer	162	6.2%
Prostate Cancer	123	4.7%
Liver Cancer	288	10.9%
Stomach Cancer	224	8.5%
Lung Cancer	528	20.1%
Colorectal Cancer	463	17.6%
Pathology of Breast Cancers (*n* = 2785) *		
Breast cancer histology		
Ductal	1872	69.7%
In situ	465	17.3%
Others	349	13.0%
Grade		
1	301	16.0%
2	858	45.6%
3	722	38.4%
Molecular Subtype		
TNBC	430	20.0%
HER2+	162	7.5%
ER/PR+	1525	70.8%
Stage		
0	483	18.6%
1	954	36.8%
2	766	29.6%
3	289	11.1%
4	100	3.9%
Pathology of Ovarian Cancers (*n* = 526)		
Ovarian cancer histology		
Epithelial	471	96.3%
Germ Cell	7	1.4%
Stromal	5	1.0%
Mixed	6	1.2%
Not stated	37	-
Grade		
1	51	11.1%
2	107	23.4%
3	294	64.2%
Mixed	6	1.3%
Not stated	68	-
Stage		
1	201	42.4%
2	59	12.4%
3	157	33.1%
4	57	12.0%
Not stated	52	-

* 552 bilateral breast cancers. TNBC: triple-negative breast cancer; HER2: human epidermal growth factor receptor 2; ER: estrogen receptors; PR: progesterone receptors.

**Table 2:** Characteristics and mutation frequencies of *PALB2* and *BRCA1/2*.

No. of Probands	*n* = 39		*n* = 173		*n* = 2 04		*n* = 377		*n* = 2214		*p*-Value			
	*PALB2*+		*BRCA1*+		*BRCA2*+		*BRCA*+		Negative		*PALB2*+ vs. *BRCA1*+	*PALB2*+ vs. *BRCA2*+	*PALB2*+ vs. *BRCA*+	*PALB2*+ vs. Negative
	n	%	n	%	n	%	n	%	n	%				
Breast cancer first DX														
Mean/Median (Range)	43.5/39 (24–69)		41.0/40 (22–73)		43.0/42 (21–73)		42.2/41 (21–73)		45.7/43 (8–88)		0.466	0.616	0.984	0.227
Ovarian cancer first DX														
Mean/Median (Range)	53.6/59 (36–65)		51.4/51 (17–85)		53.4/53 (31–74)		52.1/51 (17–85)		47.3/48 (9–79)		NA *	NA *	NA *	NA *
Gender														
Female	39	100.0%	173	100.0%	190	93.1%	363	96.3%	2161	97.6%	1.000	0.135	0.381	1.000
Male	0	0.0%	0	0.0%	14	6.9%	14	3.7%	53	2.4%				
Personal cancer of breast or ovarian cancer														
Breast	34	87.2%	93	53.8%	163	79.9%	256	67.9%	1815	82.0%	<0.001	0.749	0.050	0.356
Breast and Ovarian	2	5.1%	34	19.7%	16	7.8%	50	13.3%	75	3.4%				
Ovarian	3	7.7%	46	26.6%	25	12.3%	71	18.8%	324	14.6%				
Multiple cancer														
Yes	4	10.3%	39	22.5%	29	14.2%	68	18.0%	279	12.6%	0.121	0.618	0.272	0.811
No	35	89.7%	134	77.5%	175	85.8%	309	82.0%	1935	87.4%				
Bilateral cancer														
Yes	10	27.8%	43	33.9%	54	30.2%	97	31.7%	444	23.5%	0.550	0.844	0.707	0.553
No	26	72.2%	84	66.1%	125	69.8%	209	68.3%	1446	76.5%				
Multiple personal cancers ^														
Breast + Ovarian	1	2.6%	25	14.5%	14	6.9%	39	10.3%	59	2.7%	0.055	0.477	0.155	1.000
Breast + Colorectal	1	2.6%	2	1.2%	2	1.0%	4	1.1%	18	0.8%	0.458	0.410	0.390	0.283
Breast + Corpus uteri/uterus	1	2.6%	2	1.2%	1	0.5%	3	0.8%	19	0.9%	0.458	0.296	0.326	0.296
Ovarian + Corpus uteri/uterus	1	2.6%	1	0.6%	2	1.0%	3	0.8%	69	3.1%	0.335	0.410	0.326	1.000
Family history of cancers in 1st and 2nd degree														
Breast Cancer	19	48.7%	94	54.3%	128	62.7%	222	58.9%	710	32.1%	0.595	0.110	0.236	0.037
Ovarian Cancer	0	0.0%	49	28.3%	25	12.3%	74	19.6%	87	3.9%	<0.001	0.018	0.001	0.401
Prostate Cancer	6	15.4%	3	1.7%	24	11.8%	27	7.2%	90	4.1%	0.001	0.594	0.109	0.005
Liver Cancer	4	10.3%	19	11.0%	31	15.2%	50	13.3%	234	10.6%	1.000	0.618	0.803	1.000
Stomach Cancer	5	12.8%	29	16.8%	21	10.3%	50	13.3%	169	7.6%	0.636	0.581	1.000	0.221
Lung Cancer	6	15.4%	33	19.1%	55	27.0%	88	23.3%	434	19.6%	0.819	0.159	0.318	0.684
Colorectal Cancer	6	15.4%	28	16.2%	37	18.1%	65	17.2%	392	17.7%	1.000	0.821	1.000	0.834

Remarks: cases with double mutation of *BRCA1* and *PALB2* were excluded from the above table; * *p*-value calculations were limited by sample size; ^ only listed the multiple cancer cases with mutation in *PABL2*.

**Table 3:** Pathological characteristics and mutation frequencies of *PALB2* and *BRCA1/2*.

No. of Tumors	*n* = 46		*n* = 170		*n* = 233		*n* = 403		*n* = 2234		*p*-Value			
	*PALB2*+		*BRCA1*+		*BRCA2*+		*BRCA*+		Negative		*PALB2*+ vs. *BRCA1*+	*PALB2*+ vs. *BRCA2*+	*PALB2*+ vs. *BRCA*+	*PALB2*+ vs. Negative
	n	%	n	%	n	%	n	%	n	%				
Breast histology														
Ductal	34	81.0%	132	80.0%	156	69.0%	288	73.7%	1548	68.8%	0.124	0.369	0.507	0.305
In situ	5	11.9%	8	4.8%	40	17.7%	48	12.3%	412	18.3%				
Other	3	7.1%	25	15.2%	30	13.3%	55	14.1%	291	12.9%				
Grade (invasive grade)														
1	1	3.1%	5	3.9%	6	4.1%	11	4.0%	289	18.4%	<0.001	0.779	0.067	0.048
2	19	59.4%	30	23.3%	75	51.0%	105	38.0%	733	46.7%				
3	12	37.5%	94	72.9%	66	44.9%	160	58.0%	549	34.9%				
ER														
Positive	32	84.2%	46	29.1%	178	84.8%	224	60.9%	1517	71.9%	<0.001	1.000	0.004	0.103
Negative	6	15.8%	112	70.9%	32	15.2%	144	39.1%	592	28.1%				
PR														
Positive	23	60.5%	29	18.8%	141	68.4%	170	47.2%	1250	60.0%	<0.001	0.352	0.128	1.000
Negative	15	39.5%	125	81.2%	65	31.6%	190	52.8%	832	40.0%				
HER2														
Positive	4	13.8%	9	6.6%	36	20.6%	45	14.4%	456	25.5%	0.246	0.461	1.000	0.197
Negative	25	86.2%	128	93.4%	139	79.4%	267	85.6%	1331	74.5%				
Molecular subtype (invasive tumor only)														
TNBC	4	11.4%	91	60.7%	21	11.5%	112	33.6%	314	17.6%	<0.001	0.730	0.027	0.338
Her2+	1	2.9%	4	2.7%	5	2.7%	9	2.7%	152	8.5%				
ER/PR+	29	82.9%	46	30.7%	155	84.7%	201	60.4%	1295	72.5%				
Stage														
0	3	7.7%	15	9.2%	41	19.0%	56	14.8%	424	19.5%	0.160	0.145	0.270	0.104
1	13	33.3%	77	47.2%	68	31.5%	145	38.3%	795	36.6%				
2	18	46.2%	60	36.8%	67	31.0%	127	33.5%	620	28.5%				
3	3	7.7%	10	6.1%	33	15.3%	43	11.3%	243	11.2%				
4	2	5.1%	1	0.6%	7	3.2%	8	2.1%	90	4.1%				
Ovarian histology														
Epithelial	3	100.0%	76	100.0%	36	97.3%	112	99.1%	355	95.4%	NA *	NA *	NA *	NA *
Germ cell	0	0.0%	0	0.0%	0	0.0%	0	0.0%	7	1.9%				
Stromal	0	0.0%	0	0.0%	0	0.0%	0	0.0%	5	1.3%				
Mixed	0	0.0%	0	0.0%	1	2.7%	1	0.9%	5	1.3%				
Not stated	2	-	4	-	4	-	8	-	27	-				

Remarks: cases with double mutation of *BRCA1* and *PALB2* were excluded from above calculation; * *p*-value calculations were limited by sample size.

**Table 4:** Germline heterozygous variants identified in *PALB2*.

HVGS	Frequency	Type of Mutation	Novel/ Reference
c.15delC; p.Lys7Serfs*11	1	Frameshift termination	[18]
c.181C>T; p.Gln61*	1	Nonsense	Yes
c.211+1G>A	3	Splice site mutation	[19]
Deletion exon 4-6; c.212-712_2587-888del9762; p.Pro72Serfs*20	1	Large deletion	Yes
c.444delG; p.Lys149Serfs*28	1	Frameshift termination	[20]
c.448C>T; p.Gln150*	1	Nonsense	Yes
c.839delA; p.Asn280Thrfs*8	1	Frameshift termination	[21]
c.1038delA; p.Glu347Asnfs*9	1	Frameshift termination	Yes
c.1048C>T; p.Gln350*	1	Nonsense	[22]
c.1059delA; p.Lys353Asnfs*3	5	Frameshift termination	[23]
c.1240C>T; p.Arg414*	1	Nonsense	[24]
c.1446delC; p.Ser483Hisfs*2	1	Frameshift termination	[18]
c.1592delT; p.Leu531Cysfs*30	2	Frameshift termination	[25]
c.1914dupT; p.Glu639*	1	Nonsense	Yes
c.2016dupA; p.Glu673Argfs*42	1	Frameshift termination	Yes
c.2108T>G; p.Leu703*	7	Nonsense	[26]
c.2219_2220delAA; p.Gln740Argfs*4	1	Frameshift termination	[27]
c.2594C>G; p.Ser865*	1	Nonsense	[28]
c.2760dupA; p.Gln921Thrfs*7	1	Frameshift termination	[29]
c.2968G>T; p.Glu990*	3	Nonsense	[30]
c.3114-1G>A; r.3114_3350del237; p.Asn1039_Arg1117del79	1	Splice site mutation	[31]
c.3201+2T>C; r.3114_3350del237; p.Asn1039_Arg1117del79	1	Splice site mutation	Yes
c.3256C>T; p.Arg1086*	1	Nonsense	[32]
c.3507_3508delTC; p.His1170Phefs*19	1	Frameshift termination	[33]

## Data Availability

The dataset supporting the conclusions of this article is included within the article and its additional files. Mutated sequences (except those mutations of structural rearrangement, which required further investigation) have been submitted to Genbank.

## Supplementary Materials

The following are available online at https://www.mdpi.com/article/10.3390/cancers13164195/s1, Supplementary Figure S1a. Disease-free survival of PALB2 mutation carriers versus BRCA1 mutation carriers. Supplementary Figure S1b. Disease-free survival of PALB2 mutation carriers versus mutation BRCA2 mutation carriers. Supplementary Figure S1c. Disease-free survival of PALB2 mutation carriers versus negative mutation non-carriers. Supplementary Figure S2a. Overall survival of PALB2 mutation carriers versus BRCA1 mutation carriers. Supplementary Figure S2b. Overall survival of PALB2 mutation carriers versus BRCA2 mutation carriers. Supplementary Figure S2c. Overall survival of PALB2 mutation carriers versus mutation negative non-carriers.

## References

[1] Antoniou A.C., Casadei S., Heikkinen T., Barrowdale D., Pylkäs K., Roberts J., Lee A., Subramanian D., De Leeneer K., Fostira F. (2014). Breast cancer risk in families with mutations in PALB2. N. Engl. J. Med..

[2] Cao A.Y., Huang J., Hu Z., Li W.F., Ma Z.L., Tang L.L., Zhang B., Su F.X., Zhou J., Di G.H. (2009). The prevalence of PALB2 germline mutations in BRCA1/BRCA2 negative Chinese women with early onset breast cancer or affected relatives. Breast Cancer Res. Treat..

[3] Yang X., Leslie G., Doroszuk A., Schneider S., Allen J., Decker B., Dunning A.M., Redman J., Scarth J., Plaskocinska I. (2020). Cancer Risks Associated with Germline *PALB2* Pathogenic Variants: An International Study of 524 Families. J. Clin. Oncol..

[4] Erkko H., Xia B., Nikkilä J., Schleutker J., Syrjäkoski K., Mannermaa A., Kallioniemi A., Pylkäs K., Karppinen S.M., Rapakko K. (2007). A recurrent mutation in PALB2 in Finnish cancer families. Nature.

[5] Ece Solmaz A., Yeniay L., Gökmen E., Zekioğlu O., Haydaroğlu A., Bilgen I., Özkınay F., Onay H. (2021). Clinical Contribution of Next-Generation Sequencing Multigene Panel Testing for BRCA Negative High-Risk Patients with Breast Cancer. Clin Breast Cancer..

[6] Evans M.K., Longo D.L. (2014). PALB2 mutations and breast-cancer risk. N. Engl. J. Med..

[7] Slater E.P., Langer P., Niemczyk E., Strauch K., Butler J., Habbe N. (2010). PALB2 mutations in European familial pancreatic cancer families. Clin. Genet..

[8] Reid S., Schindler D., Hanenberg H., Barker K., Hanks S., Kalb R., Neveling K., Kelly P., Seal S., Freund M. (2007). Biallelic mutations in PALB2 cause Fanconi anemia subtype FA-N and predispose to childhood cancer. Nat. Genet..

[9] Tischkowitz M., Xia B., Sabbaghian N., Reis-Filho J.S., Hamel N., Li G., van Beers E.H., Li L., Khalil T., Quenneville L.A. (2007). Analysis of PALB2/FANCN-associated breast cancer families. Proc. Natl. Acad. Sci. USA.

[10] Deng M., Chen H.H., Zhu X., Luo M., Zhang K., Xu C.J., Hu K.M., Cheng P., Zhou J.J., Zheng S. (2019). Prevalence and clinical outcomes of germline mutations in BRCA1/2 and PALB2 genes in 2769 unselected breast cancer patients in China. Int. J. Cancer..

[11] Heikkinen T., Kärkkäinen H., Aaltonen K., Milne R.L., Heikkilä P., Aittomäki K., Blomqvist C., Nevanlinna H. (2009). The breast cancer susceptibility mutation PALB2 1592delT is associated with an aggressive tumor phenotype. Clin. Cancer Res..

[12] Southey M.C., Teo Z.L., Dowty J.G., Odefrey F.A., Park D.J., Tischkowitz M., Sabbaghian N., Apicella C., Byrnes G.B., Winship I. (2010). A PALB2 mutation associated with high risk of breast cancer. Breast Cancer Res..

[13] Rahman N., Seal S., Thompson D., Kelly P., Renwick A., Elliott A., Reid S., Spanova K., Barfoot R., Chagtai T. (2007). PALB2, which encodes a BRCA2 interacting protein, is a breast cancer susceptibility gene. Nat. Genet..

[14] Kwong A., Shin V.Y., Chen J., Cheuk I.W.Y., Ho C.Y.S., Au C.H., Chan K., Ngan H., Chan T.L., Ford J.M. (2020). Germline Mutation in 1338 BRCA-Negative Chinese Hereditary Breast and/or Ovarian Cancer Patients: Clinical Testing with a Multigene Test Panel. J. Mol. Diagn..

[15] Wu Y., Ouyang T., Li J., Wang T., Fan Z., Fan T., Lin B., Xu Y., Xie Y. (2020). Spectrum and clinical relevance of PALB2 germline mutations in 7657 Chinese BRCA1/2-negative breast cancer patients. Breast Cancer Res. Treat..

[16] Isaac D., Karapetyan L., Tamkus D. (2018). Association of germline PALB2 mutation and response to platinum-based chemotherapy in metastatic breast cancer: A case series. JCO Precis. Oncol..

[17] Kuemmel S., Harrach H., Schmutzler R.K., Kostara A., Ziegler-Löhr K., Dyson M.H., Chiari O., Reinisch M. (2020). Olaparib for metastatic breast cancer in a patient with a germline *PALB2* variant. NPJ Breast Cancer.

[18] Dicks E., Song H., Ramus S.J., Oudenhove E.V., Tyrer J.P., Intermaggio M.P., Kar S., Harrington P., Bowtell D.D., Group A.S. (2017). Germline whole exome sequencing and large-scale replication identifies FANCM as a likely high grade serous ovarian cancer susceptibility gene. Oncotarget.

[19] Ng P.S., Boonen R.A., Wijaya E., Chong C.E., Sharma M., Knaup S., Mariapun S., Ho W.K., Lim J., Yoon S.Y. (2021). Characterisation of protein-truncating and missense variants in PALB2 in 15 768 women from Malaysia and Singapore. J. Med. Genet.

[20] NM_024675.3(PALB2):c.444delG (p.Lys149Serfs). https://www.ncbi.nlm.nih.gov/clinvar/RCV000576472.

[21] NM_024675.3(PALB2):c.839del (p.Asn280fs). https://www.ncbi.nlm.nih.gov/clinvar/variation/480243/.

[22] NM_024675.3(PALB2):c.1048C>T (p.Gln350Ter). https://www.ncbi.nlm.nih.gov/clinvar/variation/410191/.

[23] NM_024675.3(PALB2):c.1059del (p.Lys353fs). https://www.ncbi.nlm.nih.gov/clinvar/variation/182745/.

[24] NM_024675.4(PALB2):c.1240C>T (p.Arg414Ter). https://www.ncbi.nlm.nih.gov/clinvar/variation/128117/.

[25] NM_024675.3(PALB2):c.1592del (p.Leu531fs). https://www.ncbi.nlm.nih.gov/clinvar/variation/126609/.

[26] NM_024675.3(PALB2):c.2108T>G (p.Leu703Ter) AND Familial cancer of breast. https://www.ncbi.nlm.nih.gov/clinvar/68467629/.

[27] NM_024675.3(PALB2):c.444del (p.Lys149fs) AND Familial cancer of breast. https://www.ncbi.nlm.nih.gov/clinvar/68737907/.

[28] NM_024675.3(PALB2):c.2594C>G (p.Ser865Ter). https://www.ncbi.nlm.nih.gov/clinvar/variation/241547/.

[29] Ng P.S., Pan J.W., Ahmad Zabidi M.M., Rajadurai P., Yip C.H., Reuda O.M., Dunning A.M., Antoniou A.C., Easton D.F., Caldas C. (2021). Characterisation of PALB2 tumours through whole-exome and whole-transcriptomic analyses. NPJ Breast Cancer.

[30] NM_024675.3(PALB2):c.2968G>T (p.Glu990Ter). https://www.ncbi.nlm.nih.gov/clinvar/variation/231227/.

[31] NM_024675.3(PALB2):c.3114-1G>A. https://www.ncbi.nlm.nih.gov/clinvar/variation/265552/.

[32] NM_024675.3(PALB2):c.3256C>T (p.Arg1086Ter). https://www.ncbi.nlm.nih.gov/clinvar/variation/126729/.

[33] NM_024675.4(PALB2):c.3507_3508del (p.His1170fs). https://www.ncbi.nlm.nih.gov/clinvar/variation/140978/.

[34] Cybulski C., Kluźniak W., Huzarski T., Wokołorczyk D., Kashyap A., Jakubowska A., Szwiec M., Byrski T., Dębniak T., Górski B. (2015). Clinical outcomes in women with breast cancer and a PALB2 mutation: A prospective cohort analysis. Lancet Oncol..

[35] Tung N., Lin N.U., Kidd J., Allen B.A., Singh N., Wenstrup R.J., Hartman A.R., Winer E.P., Garber J.E. (2016). Frequency of germline mutations in 25 cancer susceptibility genes in a sequential series of patients with breast cancer. J. Clin. Oncol..

[36] Zhou J., Wang H., Fu F., Li Z., Feng Q., Wu W., Liu Y., Wang C., Chen Y. (2020). Spectrum of PALB2 germline mutations and characteristics of PALB2-related breast cancer: Screening of 16,501 unselected patients with breast cancer and 5890 controls by next-generation sequencing. Cancer..

[37] Castéra L., Harter V., Muller E., Krieger S., Goardon N., Ricou A., Rousselin A., Paimparay G., Legros A., Bruet O. (2018). Landscape of pathogenic variations in a panel of 34 genes and cancer risk estimation from 5131 HBOC families. Genet. Med..

[38] Dorling L., Carvalho S., Allen J., González-Neira A., Luccarini C., Wahlström C., Pooley K.A., Parsons M.T., Fortuno C., Breast Cancer Association Consortium (2021). Breast Cancer Risk Genes—Association Analysis in More than 113,000 Women. N. Engl. J. Med..

[39] Erkko H., Dowty J.G., Nikkilä J., Syrjäkoski K., Mannermaa A., Pylkäs K., Southey M.C., Holli K., Kallioniemi A., Jukkola-Vuorinen A. (2008). Penetrance analysis of the PALB2 c.1592delT founder mutation. Clin. Cancer Res..

[40] Kluska A., Balabas A., Piatkowska M., Czarny K., Paczkowska K., Nowakowska D., Mikula M., Ostrowski J. (2017). PALB2 mutations in BRCA1/2-mutation negative breast and ovarian cancer patients from Poland. BMC Med. Genomics..

[41] Foules W.D., Ghadirian P., Akbari M.R., Hamel N., Giroux S., Sabbaghian N., Darnel A., Royer R., Poll A., Fafard E. (2007). Identification of a novel truncating PALB2 mutation and analysis of its contribution to early-onset breast cancer in French-Canadian women. Breast Cancer Res..

[42] Pern F., Bogdanova N., Schürmann P., Lin M., Ay A., Länger F., Hillemanns P., Christiansen H., Park-Simon T.W., Dörk T. (2021). Mutation analysis of BRCA1, BRCA2, PALB2 and BRD7 in a hospital-based series of German patients with triple-negative breast cancer. PLoS ONE.

[43] Cote S., Arcand S.L., Royer R., Nolet S., Mes-Masson A.M., Ghadirian P., Foulkes W.D., Tischkowitz M., Narod S.A., Provencher D. (2012). The BRCA2 c.9004G > A (E2002K) variant is likely pathogenic and recurs in breast and/or ovarian cancer families of French Canadian descent. Breast Cancer Res. Treat..

[44] Yoshikiyo K., Kratz K., Hirota K., Nishihara K., Takata M., Kurumizaka H., Horimoto S., Takeda S., Jiricny J. (2010). KIAA1018/FAN1 nuclease protects cells against genomic instability induced by interstrand cross-linking agents. Proc. Natl. Acad. Sci. USA.

[45] Gruber J.J., Afghani A., Hatton A., Scott D., McMillan A., Ford J.M., Telli M.L. (2019). Talazoparib beyond BRCA: A phase II trial of talazoparib monotherapy in *BRCA1* and *BRCA2* wild-type patients with advanced HER2-negative breast cancer or other solid tumors with a mutation in homologous recombination (HR) pathway genes. J. Clin. Oncol..

[46] Tung N.M., Robson M.E., Ventz S., Santa-Maria C.A., Nanda R., Marcom P.K., Shah P.D., Ballinger T.J., Yang E.S., Vinayak S. (2020). TBCRC 048: Phase II Study of Olaparib for Metastatic Breast Cancer and Mutations in Homologous Recombination-Related Genes. J. Clin. Oncol..

[47] Grellety T., Peyraud F., Sevenet N., Tredan O., Dohollou N., Barouk-Simonet E., Kind M., Longy M., Blay J.Y., Italiano A. (2020). Dramatic response to PARP inhibition in a PALB2-mutated breast cancer: Moving beyond BRCA. Ann. Oncol..

[48] (2021). USA National Library of Medicine. https://clinicaltrials.gov/ct2/show/NCT04756765?term=PALB2&cond=breast+cancer&draw=2&rank=2.

[49] (2021). NCCN Clinical Practice Guidelines in Oncology (NCCN Guidelines). https://www2.tri-kobe.org/nccn/guideline/gynecological/english/genetic_familial.pdf.

[50] Tischkowitz M., Balmaña J., Foulkes W.D., James P., Ngeow J., Schmutzler R., Voian N., Wick M.J., Stewart D.R., Pal T. (2021). ACMG Professional Practice and Guidelines Committee. Management of individuals with germline variants in *PALB2*: A clinical practice resource of the American College of Medical Genetics and Genomics (ACMG). Genet. Med..

[51] Kotsopoulos J., Sopik V., Rosen B., Fan I., McLaughlin J.R., Risch H., Sun P., Narod S.A., Akbari M.R. (2017). Frequency of germline PALB2 mutations among women with epithelial ovarian cancer. Fam. Cancer..

[52] Janssen B., Bellis S., Koller T., Tischkowitz M., Liau S.S. (2020). A systematic review of predicted pathogenic PALB2 variants: An analysis of mutational overlap between epithelial cancers. J. Hum. Genet..

[53] Kwong A., Shin V.Y., Au C.H., Law F.B.F., Ho D.N., Wong A.T.C., Lau S.S., To R.M., Choy G., Ford J.M. (2016). Detection of germline mutation in hereditary breast and/ovarian cancers by next-generation sequencing on a four gene panel. J. Mol. Diagn..

[54] Neben C.L., Zimmer A.D., Stedden W., van den Akker J., O’Connor R., Chan R.C., Chen E., Tan Z., Leon A., Ji J. (2019). Multi-Gene Panel Testing of 23,179 Individuals for Hereditary Cancer Risk Identifies Pathogenic Variant Carriers Missed by Current Genetic Testing Guidelines. J. Mol. Diagn..

[55] (2015). The 1000 Genomes Project Consortium: A global reference for human genetic variation. Nature.

