# Supplementary material for: Germline PALB2 Mutation in High-Risk Chinese Breast and/or Ovarian Cancer Patients
Source: Cancers (Basel). 2021 Aug 20;13(16):4195. doi: 10.3390/cancers13164195 (PMC8394494; doi:10.3390/cancers13164195)
Supplement: Supplementary file 1 [file cancers-13-04195-s001.zip › Supplementary Figure 1b.pdf]

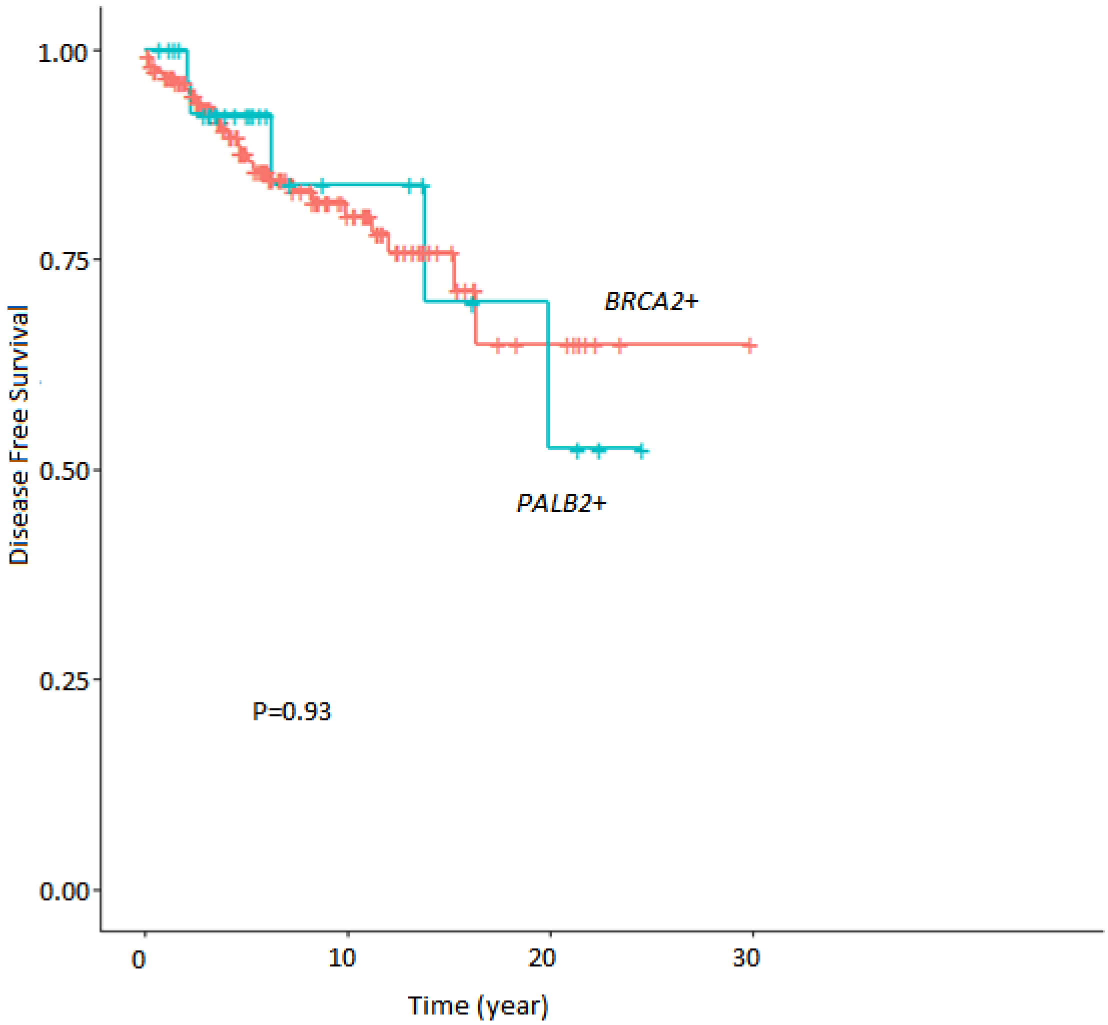

Supplementary Figure 1b. Disease free survival of *PALB2* mutation carriers versus mutation *BRCA2* mutation carriers. Disease free survival is defined at the time from diagnosis to first relapse or death. Stage IV cases were excluded. For bilateral cases, if the onset time of 2nd primary was >90 days after 1st primary and before any relapse of 1st primary, the case was treated as censored at the time of 2nd primary diagnosis.
