# Supplementary material for: Germline PALB2 Mutation in High-Risk Chinese Breast and/or Ovarian Cancer Patients
Source: Cancers (Basel). 2021 Aug 20;13(16):4195. doi: 10.3390/cancers13164195 (PMC8394494; doi:10.3390/cancers13164195)
Supplement: Supplementary file 1 [file cancers-13-04195-s001.zip › Supplementary Figure 2c.pdf]

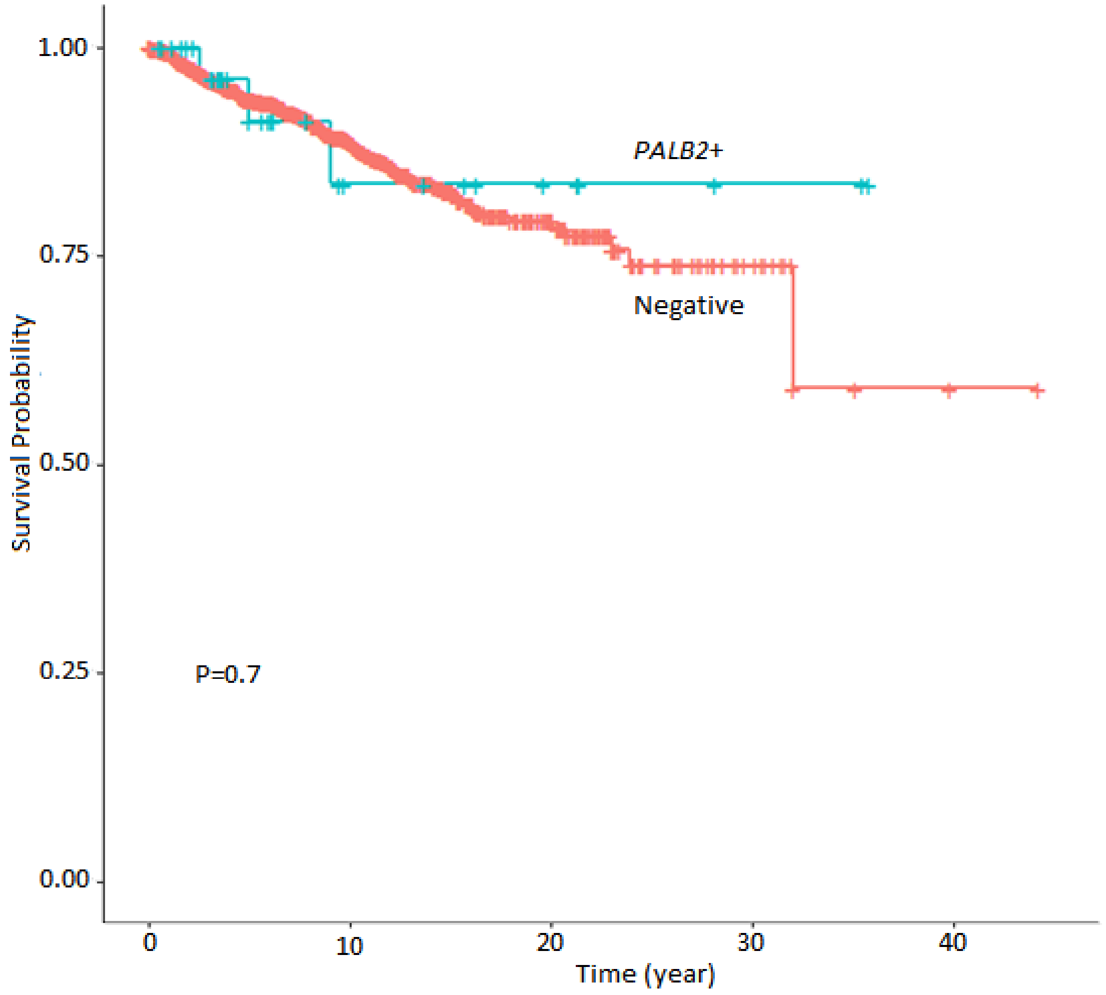

Supplementary Figure 2c. Overall survival of *PALB2* mutation carriers versus mutation negative non-carriers.
